# Diastolic function assessment with four-dimensional flow cardiovascular magnetic resonance using automatic deep learning E/A ratio analysis

**DOI:** 10.1016/j.jocmr.2024.101042

**Published:** 2024-03-30

**Authors:** Federica Viola, Mariana Bustamante, Ann Bolger, Jan Engvall, Tino Ebbers

**Affiliations:** a Division of Diagnostics and Specialist Medicine, Department of Health, Medicine and Caring Sciences, Linköping University, Linköping, Sweden; b Center for Medical Image Science and Visualization (CMIV), Linköping University, Linköping, Sweden; c deCODE Genetics/Amgen Inc., Reykjavik, Iceland; d Department of Medicine, University of California San Francisco, San Francisco, CA, United States; e Department of Clinical Physiology in Linköping, and Department of Health, Medicine and Caring Sciences, Linköping University, Linköping, Sweden

**Keywords:** 4D Flow CMR, Diastolic function, EA ratio, Deep learning

## Abstract

**Background:**

Diastolic left ventricular (LV) dysfunction is a powerful contributor to the symptoms and prognosis of patients with heart failure. In patients with depressed LV systolic function, the E/A ratio, the ratio between the peak early (E) and the peak late (A) transmitral flow velocity, is the first step to defining the grade of diastolic dysfunction. Doppler echocardiography (echo) is the preferred imaging technique for diastolic function assessment, while cardiovascular magnetic resonance (CMR) is less established as a method. Previous four-dimensional (4D) Flow-based studies have looked at the E/A ratio proximal to the mitral valve, requiring manual interaction. In this study, we compare an automated, deep learning-based and two semi-automated approaches for 4D Flow CMR-based E/A ratio assessment to conventional, gold-standard echo-based methods.

**Methods:**

Ninety-seven subjects with chronic ischemic heart disease underwent a cardiac echo followed by CMR investigation. 4D Flow-based E/A ratio values were computed using three different approaches; two semi-automated, assessing the E/A ratio by measuring the inflow velocity (MVvel) and the inflow volume (MVflow) at the mitral valve plane, and one fully automated, creating a full LV segmentation using a deep learning-based method with which the E/A ratio could be assessed without constraint to the mitral plane (LVvel).

**Results:**

MVvel, MVflow, and LVvel E/A ratios were strongly associated with echocardiographically derived E/A ratio (R^2^ = 0.60, 0.58, 0.72). LVvel peak E and A showed moderate association to Echo peak E and A, while MVvel values were weakly associated. MVvel and MVflow EA ratios were very strongly associated with LVvel (R^2^ = 0.84, 0.86). MVvel peak E was moderately associated with LVvel, while peak A showed a strong association (R^2^ = 0.26, 0.57).

**Conclusion:**

Peak E, peak A, and E/A ratio are integral to the assessment of diastolic dysfunction and may expand the utility of CMR studies in patients with cardiovascular disease. While underestimation of absolute peak E and A velocities was noted, the E/A ratio measured with all three 4D Flow methods was strongly associated with the gold standard Doppler echocardiography. The automatic, deep learning-based method performed best, with the most favorable runtime of ∼40 seconds. As both semi-automatic methods associated very strongly to LVvel, they could be employed as an alternative for estimation of E/A ratio.

## Graphical abstract

**Figure:**
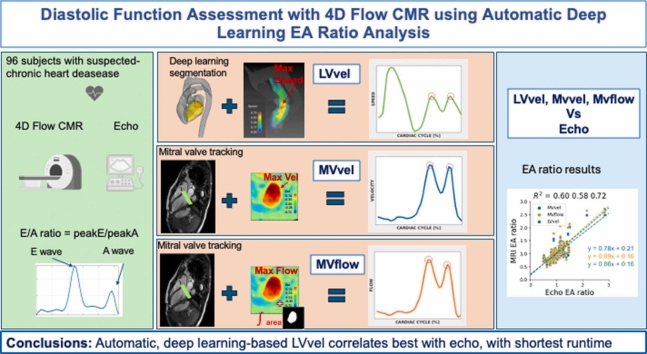

## 1 Introduction

Diastolic left ventricular (LV) dysfunction is a powerful contributor to the symptoms and prognosis of patients with heart failure. Doppler echocardiography (echo) is the preferred imaging technique for diastolic function assessment due to its accuracy, safety, availability, and cost efficiency [1]. Detection of diastolic dysfunction and elevated left atrial (LA) pressure with echo relies on a combination of measurements of velocity of the mitral inflow and the mitral annular excursion, along with signs of LA enlargement and pulmonary hypertension. In patients with depressed LV systolic function (left ventricular ejection fraction (LVEF) less than 50%, or with myocardial disease), the ratio between the peak velocities of the early (E) and late (A) mitral inflow, known as the E/A ratio, is the first step in the clinical algorithm defining the grade of diastolic dysfunction and the presence of elevated LA pressure. The accuracy of the assessment of LV systolic function, myocardial abnormalities, the mitral inflow velocities, and the E/A ratio is therefore critical when using cardiac imaging to comprehensively define ventricular function in both systole and diastole. While echo is the gold standard approach to defining diastolic function, cardiovascular magnetic resonance (CMR) is the gold standard for assessing cardiac volume, mass, and ejection fraction, and has a class I indication for patients with heart failure and poor acoustic windows [2]. However, CMR is less well established as a method for defining diastolic dysfunction. As CMR and echo are both commonly used in patients with cardiovascular disease, it is of utmost importance to make comparable measurements and to define and minimize differences between these methods.

Several feasible CMR approaches exist for assessing diastolic function, including LV and LA volume-time curve analysis, LV-LA feature tracking and tagging, and mitral inflow quantification [3]. Two-dimensional cine phase contrast CMR (2D PC-CMR) with single-direction velocity encoding (VENC) can be also used for mitral inflow quantification. With this approach, a plane is placed at the area of interest, perpendicular to the expected flow direction, to measure the time-varying inflow volume or velocities. Inflow velocities and E/A ratios derived from 2D PC-CMR flow measured at a plane located at the tips of the open mitral leaflets correlate strongly with the corresponding echo values, despite an underestimation of the absolute values of E and A [4]. This underestimation might be due to several factors, including smoothing of velocity values due to averaging over several cardiac cycles, the use of single-direction VENC, and the fixed plane position interfering with capture of the highest speed locations [5], [6], [7].

Four-dimensional cine phase contrast CMR (4D Flow), a time-resolved three-dimensional cine PC-CMR with three-directional velocity-encoding, overcomes some of the limitations of 2D PC-CMR, as it permits measurement of the velocities in any direction at any site within the acquired volume [8]. By following the mitral valve plane over the cardiac cycle, a direct measurement of the mitral flow pattern can be obtained [9]. The mitral inflow can be quantified according to volume, velocity, or even kinetic energy [10]. A recent meta-analysis demonstrated that 4D Flow was superior to 2D PC-CMR in measuring peak velocity values at multiple sites in comparison to echo [11]. An important feature of 4D Flow in the clinical setting is the ability to retrospectively position analysis planes at regions of interest to measure flow volumes through valves, vessels, and shunts. The flow across the mitral valve has been shown to be accurately measured from a plane at the annulus that tracks its position over the cardiac cycle [12].

Comparisons of 2D PC-CMR and 4D Flow measurements of clinical diastolic function parameters have demonstrated the superior correlation of 4D Flow with Doppler echo results in studies on healthy subjects [13] and in patients with mild to severe diastolic dysfunction [14]. The CMR methods have relied on manual data post-processing; however, it requires operator expertise, is time-consuming, and is associated with intra- and inter-observer variability. Automated or semi-automated methods may avoid these constraints and make the CMR assessment of E/A ratios more clinically available. In this study, we compare one automated, deep learning-based method, and two semi-automated approaches for 4D Flow CMR-based E/A ratio assessment to conventional, gold standard echo-based results.

## 2 Material and methods

### 2.1 Study population

Ninety-seven subjects with suspected chronic ischemic heart disease were recruited from a 2-year follow-up of the DOPPLER-CIP study [15], and 77 adequate data sets were analyzed. Mean age was 67.4 ± 5.8 (51−80), 21 were female. Mean weight and height were 81 ± 16.6 kg (53−114) and 1.73 ± 0.1 m (1.46–1.93), while mean systolic and diastolic pressure were 143/79 mmHg (115–205/6 ± 100). On echo, the LVEF was 58.2 ± 3.9 (44.7–66.1); two patients had LVEF less than 50% and grade 1 diastolic dysfunction. The E/A ratio was 1.0 ± 0.5 (0.5–2.8); two patients had an E/A ratio greater than 2. No patient had more than moderate valvular disease.

All subjects underwent a cardiac echo followed by an MRI investigation within 4 hours.

### 2.2 CMR acquisition and post-processing

The CMR exams were performed on a Philips 3T MRI system (Ingenia CV, Philips Healthcare, Best, The Netherlands, software release R4.1.2). The imaging protocol included a balanced steady-state free precession acquisition (bSSFP), acquired at end-expiratory breath-hold, and a free-breathing, respiratory-motion-compensated 4D Flow examination. An anterior coil consisting of 16 channels was used for the acquisition.

The cine bSSFP acquisitions consisted of a cardiac short-axis (SA) stack with acquired in-plane spatial resolution of 2 × 2 mm^2^, reconstructed to 1 × 1 mm^2^, and acquired/reconstructed through-plane resolution of 8 mm, and two-, three, and four-chamber long-axis images (2ch-, 3ch-, 4ch-images) of the left heart, with the same acquired/reconstructed in-plane resolution as the SA stack. Other scan parameters included echo time (TE) of 1.38 ms, repetition time (TR) 2.8 ms, flip angle 45°, and acquired temporal resolution 53 ms. All cine images were reconstructed to 30 time-frames.

The retrospectively cardiac-gated and respiratory navigator-gated 4D Flow MRI examination consisted of a spoiled gradient echo (SGRE) sequence. All subjects received a gadolinium contrast agent (0.2 mmol/kg, Gadovist, Bayer Healthcare, Berlin, Germany) prior to the 4D flow CMR examination. An asymmetric 4-point motion encoding scheme was used. Other scan parameters included candy cane view covering both ventricles, VENC 120 cm/s, flip angle 10°, TE 2.6 ms, TR 4.4 ms, k-space segmentation factor 3, parallel imaging (sensitivity encoding, SENSE) speed up factor 3 (in the anterior-posterior direction), acquired/reconstructed spatial resolution ∼2.8 mm^3^ isotropic voxels, and elliptical k-space acquisition. The effective temporal resolution was 52.8 ms. Typical scan time was approximately 7–8 minutes, navigator excluded, and 8–12 minutes with navigator. The respiratory navigator gating used an inner window of 4 mm for 20% of the k-space and an outer window of 15 mm.

All 4D Flow data were corrected for concomitant gradient fields on the scanner and corrected for phase wraps and background phase errors during post-processing [16], [17].

### 2.3 4D Flow-based E/A ratio

**Fig. 1:**
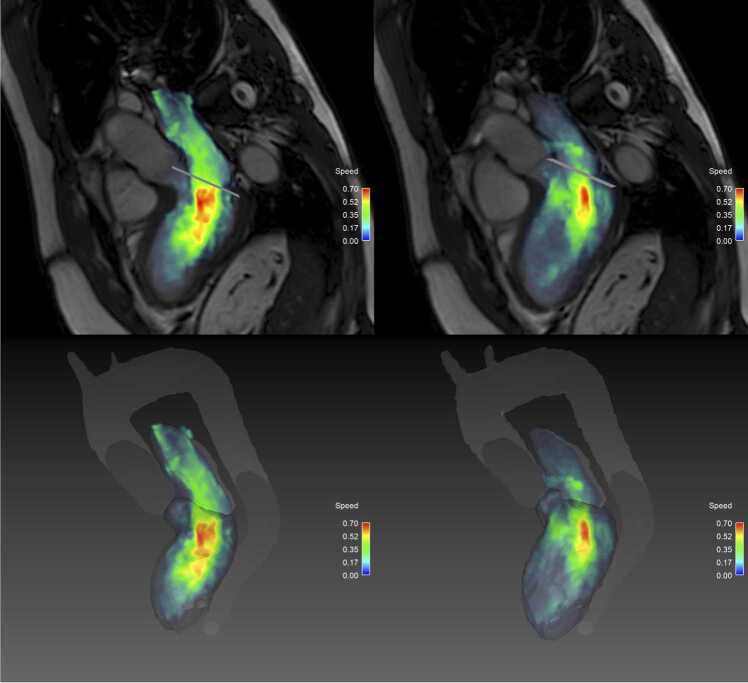
Example of the semi-automatic methods, MVvel and MVflow, and the automatic method LVvel during early and late filling. Upper row: In MVvel and MVflow, the mitral valve position was tracked from a three-chamber image, and the velocity reformatted over a plane. Lower row: In LVvel, cardiovascular 4D deep learning-based segmentations were created and the left ventricular mask used to determine the maximum velocity inside the left ventricle only. The speed at early (left) and late filling (right) is shown as the maximum intensity projection over the three-chamber view. MVvel, mitral inflow velocity; MVflow, mitral inflow volume; LVvel, left ventricular velocity.

4D Flow-based E/A ratio values were computed using three different approaches. Two semi-automated methods were used to assess the inflow at the level of the mitral valve plane (Fig. 1, upper row), one by measuring the inflow velocity (MVvel) and the other by measuring the inflow volume (MVflow). The third approach (Fig. 1, lower row) was completely automated and created a full LV segmentation within which the maximum inflow speed could be measured during early and late diastole without constraint to the mitral plane (left ventricular velocity (LVvel)).

MVvel and MVflow: Using 4D Flow data, E/A ratios were assessed from the velocity and the inflow volume curves taken from the time-varying position of the mitral valve plane. The initial mitral valve position was manually identified on a 3ch image at end-diastole and automatically tracked over the cardiac cycle using in-house software following a previously published approach [18], [19]. Optimal tracking accuracy was obtained by combining the tracking positions from tracking one cardiac cycle forward and one cardiac cycle backward in time. Weighted averaging was used, with weights decreasing from the starting time point. The velocities were thus reformatted over the tracked 2D plane and the inflow area was segmented over the cardiac cycle. The through-plane velocity was computed, corrected for mitral valve movement, and median-filtered (3 × 3 kernel). The two highest peaks in the maximum velocity curve were then selected as E (the earlier peak) and A (the later peak) for MVvel. The peak selection was performed automatically: two prominent peaks were recognizable between end-systole to end-diastole (end of cardiac cycle). The peak selection was double checked manually for potential mistakes. In MVflow, the through-plane velocity was integrated over the segmented area, and peaks E and A defined as the highest peaks in the flow curve during diastole, in the same way as done for MVvel. Computation time for a single dataset was around 5 minutes, for each method.

LVvel: Automatic segmentations of the heart were created with a deep learning approach, which generates 4D segmentations of the cardiac chambers and main vessels [20]. The LV segmentation was used to mask the 4D Flow volume, and peak E and A speed values were identified as the highest values at any site within the LV volume during early and late diastole. Before identification of the peaks, the speed in the LV was median-filtered with a 3 × 3 × 3 kernel. The computation time for a single dataset was ∼40 seconds.

### 2.4 Data excluded from the comparison

Twenty datasets were subsequently excluded from the study. Six datasets were excluded due to a monophasic inflow curve at the MRI examination where E and A peaks could not be separately discerned due to high heart-rate (mean heart rate 92 beats per minute). Fourteen datasets were excluded due to severe artifacts in the 4D Flow magnitude images. Causes of these were metal artifacts due to post-operative sternal wires (n = 5), suboptimal flip angle at the time of acquisition (n = 6), or inadequate shimming (n = 3).

### 2.5 Echo acquisition

**Fig. 2:**
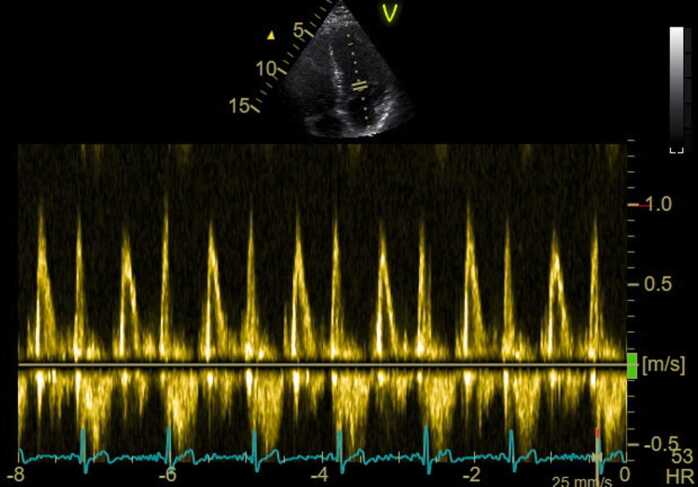
Example of E/A ratio assessment with echo, for the same patient as the one shown in Fig. 1. Parallel solid lines on the sector image indicate the manually placed location of the Doppler sample volume for velocity assessment at the level of the tips of the mitral valve leaflets. The heart rate during the examination was 53 beats per minute. HR, HeartRate.

A standard echocardiogram was obtained with the patient in the left lateral decubitus position using the following scan sequences: 2D-gray scale imaging of standard parasternal and apical views, blood Doppler imaging of left and right ventricular in- and outflows, tissue Doppler imaging of the mitral and tricuspid annuli and 3D-volume acquisitions of the left ventricle [15]. Mitral inflow velocity data were taken at the level of the mitral leaflet tips (Fig. 2).

### 2.6 Statistical analysis

Both echo and CMR data were analyzed in a blinded fashion. Linear regression and Bland-Altman analyses were performed to compare echography-based and 4D Flow-based E/A ratio, peak E and peak A values. Student t-test was used for paired comparisons. Results were considered statistically different for a p-value <0.001, with a level of significance alpha = 0.05. Association, derived from the determination coefficients, was considered weak with R^2^ ≤ 0.25, moderate 0.25 < R^2^ ≤ 0.5, strong 0.5 < R^2^ ≤ 0.8, very strong 0.8 < R^2^ ≤ 1. The Fisher’s r to z transformation was used to compare the correlation coefficients. In the Bland-Altman analysis, the percentage differences were computed as 100 * (data1 − data2)/average(data1 − data2).

## 3 Results

### 3.1 4D Flow CMR vs Echo

**Fig. 3:**
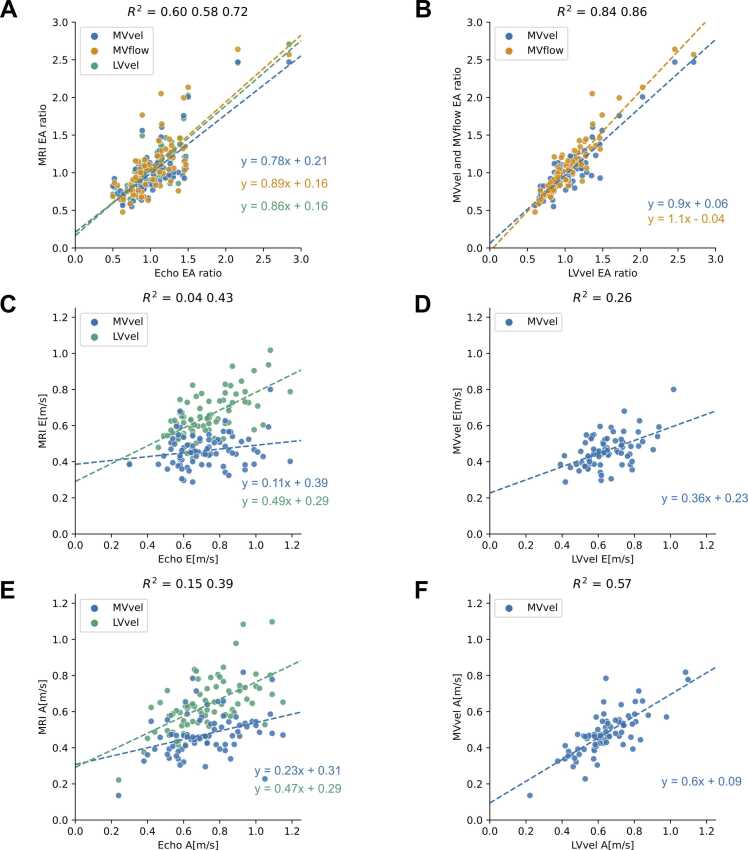
Linear regression plots. Regression lines are plotted and color-coded according to the 4D Flow CMR method. The gray line represents unity. **(A)** E/A ratio, CMR vs echo. **(B)** E/A ratio MVvel and MVflow vs LVvel. **(C)** Peak E LVvel and MVvel vs echo. **(D)** Peak-E MVvel vs LVvel. **(E)** Peak A LVvel and MVvel vs echo. **(F)** Peak A MVvel vs LVvel. CMR, cardiovascular magnetic resonance; MVvel, mitral inflow velocity; MVflow, mitral inflow volume; LVvel, left ventricular velocity.

MVvel, MVflow, and LVvel E/A ratios were strongly associated with echo E/A ratio (R^2^ = 0.60, 0.58, 0.72) (Fig. 3 A and Table 1). The correlation coefficients r were computed and compared using the Fisher’s r to z transformation, resulting in no difference between correlations (z = 1.43, 1.58, and p = 0.0764, 0.0571 for LVvel vs MVvel and LVvel vs MVflow, respectively). LVvel peaks E and A showed moderate association to echo peaks E and A, while MVvel values were weakly associated (LVvel R^2^ = 0.43, 0.39, MVvel R^2^ = 0.04, 0.15) (Fig. 3C and E). All 4D Flow-derived peak E and A values were lower than echo values, with the least underestimation by LVvel values (p < 0.001).

**Table 1:** The linear regression and Bland-Altman coefficients of EA ratio, peak E, and peak A.

		Metric	R^2^	β1	β0	Mean	Mean − 2SD	Mean + 2SD
LVvel	vs echo	E/A ratio	0.72	0.86	0.16	−0.02	−0.40	0.36
MVvel			0.60	0.78	0.21	0.02	−0.44	0.48
MVflow			0.58	0.89	0.16	−0.05	−0.57	0.48
LVvel	vs echo	Peak E	0.43	0.49	0.29	0.07	−0.17	0.32
MVvel			0.04	0.11	0.39	0.26	−0.08	0.59
LVvel	vs echo	Peak A	0.39	0.47	0.29	0.09	−0.21	0.40
MVvel			0.15	0.23	0.31	0.25	−0.11	0.62
MVvel	vs LVvel	E/A ratio	0.84	0.90	0.06	0.04	−0.24	0.33
MVflow			0.86	1.06	−0.04	−0.02	−0.32	0.27
MVvel	vs LVvel	Peak E	0.26	0.36	0.23	0.18	−0.03	0.04
		Peak A	0.57	0.60	0.09	0.16	−0.03	0.35

The linear regression model is y = β1x + β0. The mean of the differences (mean) and limits of agreement (mean + 2 SD and mean − 2 SD) are shown. LVvel, left ventricular velocity; MVvel, mitral inflow velocity; MVflow, mitral inflow volume; SD, standard deviation

**Fig. 4:**
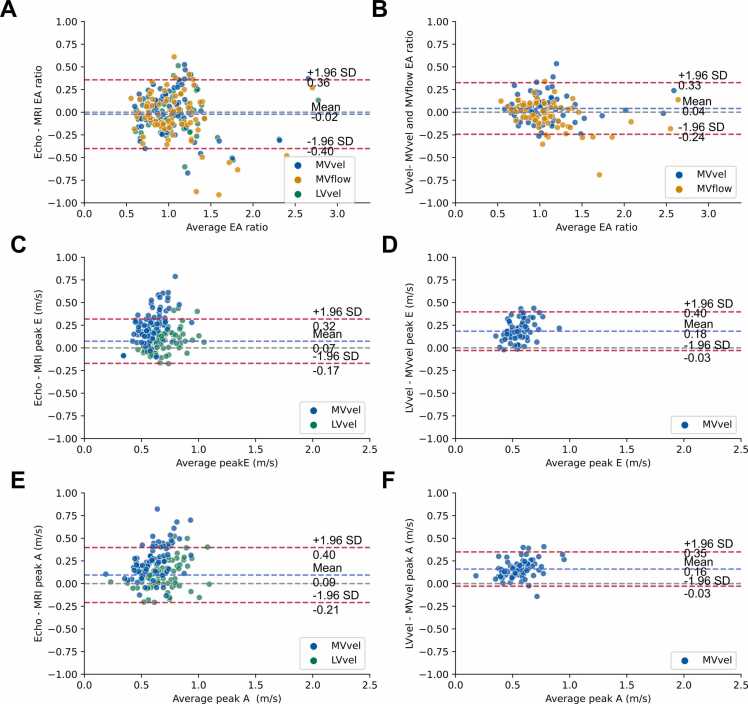
Bland-Altman plots. **(A)** E/A ratio, CMR vs echo. **(B)** E/A ratio MVvel and MVflow vs LVvel. **(C)** Peak E LVvel and MVvel vs echo. **(D)** Peak E MVvel vs LVvel. **(E)** Peak A LVvel and MVvel vs echo. **(F)** Peak A MVvel vs LVvel. The limits of agreements shown in A and C are relative to LVvel, while in B, D,and F to MVvel. CMR, cardiovascular magnetic resonance imaging; MVvel: mitral inflow velocity, MVflow, mitral inflow volume; LVvel, left ventricular velocity.

The Bland-Altman analysis yielded a mean of differences of -0.02, 0.02, and -0.05, for MVvel, MVflow, and LVvel E/A ratios vs echo E/A ratio, respectively (Fig. 4A), corresponding to a mean of percentage differences of 1.8%, −3.1%, and −2.4%. The complete set of results of the Bland-Altman analysis is shown in Table 1.

### 3.2 Semi-automated MV vs Automated LV 4D Flow MRI methods

MVvel and MVflow E/A ratios were very strongly associated with LVvel (R^2^ = 0.84, 0.86) (Fig. 3 and Table 1). MVvel peak E and A values were underestimated compared to LVvel (p < 0.001). MVvel peak E was moderately associated with LVvel, while peak A showed strong association (R^2^ = 0.26, 0.57).

Bland-Altman analysis yielded a mean of differences of 0.04 and −0.02 for MVvel and MVflow vs LVvel E/A ratio, respectively, corresponding to a mean of percentage differences of 4.1% and −0.8%. All other analysis coefficients are reported in Table 1.

## 4 Discussion

This study compares two semi-automatic and one automatic 4D flow-based method for the assessment of diastolic function parameters. Peak E, peak A, and E/A ratio are integral to the assessment of diastolic dysfunction and may expand the utility of CMR studies in patients with cardiovascular disease. While underestimation of absolute peak E and A velocities was noted, the E/A ratio measured with all three 4D Flow methods was strongly associated with the gold standard Doppler echocardiography.

Echo-based diastolic function evaluation is based on velocity data. CMR methods can provide either volume-based or velocity-based measurements; the E/A ratio can be well approximated with 4D Flow by using the time-varying mitral inflow volume measured from the mitral annulus using valve tracking [13], [14]. Accurate measurement of absolute peak E and peak A inflow velocities presents challenges for both echo and CMR methods, relating to the site of measurement, rapid acceleration, and alignment with flow. Peak inflow velocities occur at the site of the vena contracta distal to the leaflet tips; annular-focused methods have been shown, in this and prior studies, to underestimate velocities relative to vena contracta measurement sites [11], [12]. The better performance of the LVvel method, where the peak velocities are measured with three directional velocities and without constraint to location, likely reflects the ability to measure peak E and A at their maximal sites. This is particularly useful as E and A vena contracta sites are often offset, a finding that complicates echo Doppler measurements as well [7]. Temporal resolution is another important limitation, as mitral inflow and annular excursion are characterized by rapid acceleration and motion. Finally, alignment with flow is an important limitation of echo and single velocity CMR methods but is not an issue with three-directional velocity measurements as with 4D Flow.

Several prior investigations have compared 4D Flow and Doppler echocardiography diastolic parameters. In a cohort of healthy subjects, Alattar et al. compared 4D flow and echo-derived peak E, peak A, and E/A ratios using manual methods and demonstrated that taking 4D Flow volume or velocity measurements from the annulus using valve tracking or velocities from a plane at the leaflet tips correlated better with echo compared to a fixed annular plane method [13]. In MVvel and MVflow, we did not adjust the position of the analysis planes to the leaflets tips, as suggested by Alattar et al. [13], because those were not always clearly visible in the cine images, and any adjustment would have needed to be done manually, for each timeframe, for each patient. Such analysis would be difficult to apply to a large cohort. Our aim was to investigate the correlation of the semi-automatic methods to the automatic method LVvel, which in contrast is not constrained to the mitral annulus, and so is capable of capturing the highest values at the vena contracta wherever it may be located. We therefore recommend the use of the LVvel method, even if all the 4D Flow methods result in similar E/A ratio.

In patients with ischemic heart disease, Brandts et al. showed better performance of 4D flow using inflow volume with valve tracking vs 2D PC-CMR, and a strong agreement between 4D flow and echo for distinguishing subjects with severe, restrictive diastolic filling pattern (E/A ratio > 2) from subjects with less severe diastolic dysfunction [14]. In the present study of patients with ischemic heart disease, we demonstrate the feasibility of the three different methods, one completely automatic, for making diastolic inflow measurements with 4D flow.

The peak E and A velocities assessed with the 4D Flow methods underestimated echo values. A meta-analysis of studies comparing peak velocities by echo and CMR found an approximate 9% velocity underestimation using 4D flow [11]. Of the 4D methods studied here, the automatic method, LVvel, performed best in comparison with echo. LVvel-measured peak E and A velocities were moderately associated with echo values and underestimated echo values by a mean of 9.2%. Peak E and A velocities obtained with MVvel underestimated echocardiographic values, with a mean difference of 25.7 and 25.4 cm/s for E and A, respectively, which is in line with previous reported values (23.5 and 20.3 cm/s) [14]. Underestimation of peak velocities in 4D flow MRI is a known phenomenon, due to its lower temporal resolution and averaging of velocity over many cardiac cycles. An additional possible source of peak underestimation in this study is the use of a median filter, which was useful in filtering out noise and preventing isolated voxels with abnormally high values from being taken into account.

All MR methods were very strongly associated with each other for E/A ratio measurement. Peak velocities from MVvel were moderately (E peak) and strongly (A peak) associated with LVvel, although underestimated. The difference in association between E peak and A peak could be a result of the difference in LV filling mechanisms between E and A, or due to higher uncertainty in the mitral tracking during peak. The underestimation by MVvel compared to LVvel can be explained by the mitral annulus plane being located a few pixels away from the highest velocity at the vena contracta. However, the MVvel E/A ratio was not underestimated compared to LVvel, indicating that the underestimation in peak velocities is systematic, and cancels out in the ratio. The E/A ratio measured with MVflow was also very strongly correlated to LVvel E/A ratio. Thus, either of the two semi-automated methods could be employed as an alternative to LVvel for estimation of E/A ratio.

The E/A ratios from all MR methods were strongly associated with echo results, and of the three approaches, the automatic method, LVvel, showed the strongest association with echo. This method applies a deep learning-based segmentation model to create a 4D segmentation of the LV, allowing for automatic hemodynamic assessment of diastolic function. Such analysis can be run on datasets acquired routinely with 4D flow CMR, without additional human intervention. The favorable performance of this automated approach implies several advantages over semi-automated 4D flow methods as well as echo Doppler. The automated method does not require any operator expertise, is robust, and is not affected by inter- and intra-observer variability. In contrast, moderate to high inter- and intra-observer variability has frequently been reported for echo assessments of diastolic function, impacting interpretation, reproducibility, and comparisons between diastolic function assessment methods recommended by different professional societies [21], [22], [23], [24]. Finally, the automatic method is fast, with a runtime of less than a minute per patient, which implies an advantage compared to all other methods.

The algorithm-based evaluation of diastolic function using echo Doppler data relies on parameters of direct cardiac performance (inflow and annular velocities) and on associated findings (pulmonary hypertension and left atrial remodeling [1]). The use of CMR-derived results directly in a diastolic dysfunction algorithm developed for echo is unproven. In parallel logic to those guidelines, however, a patient undergoing CMR with normal LVEF, normal LV mass, and normal LA volume would be unlikely to have diastolic dysfunction. In CMR studies where the LVEF is less than 50% and/or there is evidence of myocardial abnormality or LA enlargement, an E/A ratio measured with 4D Flow CMR of less than or equal to 0.8 would suggest mild diastolic dysfunction, and an E/A ratio of >2 would suggest severe, grade 3 diastolic dysfunction with elevated left atrial pressure. Current clinical interpretation of the significance of E/A ratios between 0.8 and 2.0 relies on additional echo-based data about the degree of pulmonary hypertension, left atrial enlargement, and the ratio of peak E to annular velocity (e′). Complementary CMR parameters might be brought to bear. How well the diastolic dysfunction grade assigned using CMR-obtainable parameters would align with echocardiographic grading would require further study.

## 5 Limitations

All subjects in this study had received contrast agent before the 4D flow CMR acquisition.

The convolutional neural network used for creating the automatic segmentations was originally trained on data with contrast agent. The automatic LVvel measurement has not been tested on data without contrast agent, where segmentations might be less accurate. In this case, the network should be retrained using non-contrast data. Were this the case, however, the MVvel and MVflow assessments of the E/A ratio would remain robust, as insensitive to the 4D flow magnitude image quality.

In this study, 20 out of 97 subjects were excluded from the comparison. Of these, six were excluded due to a suboptimal setting of the flip angle due to a human error. An additional five datasets were excluded due to metal artifacts on the thorax, causing large region of signal void, and three datasets due to inadequate shimming. In six datasets, discrete E and A peaks could not be differentiated in the mitral flow curve. This phenomenon is often seen at high heart rates with echo but may be noted more often in 4D flow CMR due to its limited temporal resolution in combination with averaging over many cardiac cycles. The 4D flow CMR acquisition used in this study had a relatively low effective temporal resolution. A better temporal resolution, as recommended in the latest 4D flow consensus statement [9], is expected to result in less underestimation of E and A velocities and improve differentiation of E and A peaks in patients with high heart rates.

This study included subjects with various degrees of diastolic dysfunction, but only two had LVEF <50%, and two other subjects had E/A ratios larger than 2. One of the requirements for the subjects included in the study was to be able to perform a bicycle exercise test, and most of them were recruited from the waiting list for cardiological consultation. Both requirements tend to favor patients in relatively good health. Further tests would be needed to evaluate the methods on patients with more severe LV diastolic dysfunction. Another limitation is that all the methods have been tested on data acquired at a single center.

## 6 Conclusions

We have evaluated one automatic and two semi-automatic methods for E/A ratio assessment using 4D flow CMR data. All methods showed strong correlation with echo, and the automatic segmented LV volume method had the best agreement between echo and CMR measurements of E/A ratio, peak E and peak A velocities. Extending the CMR assessment of cardiac function to include gradation of diastolic dysfunction will augment the diagnostic and prognostic value of those studies in patients with heart disease.

## Funding

This work was funded by 10.13039/501100004359Swedish Research Council, grant number 2022-03931, the Swedish Heart and Lung Foundation, grant number 20210441, ALF Grants Region Östergötland, grant number RÖ-987498, the Sweden’s Innovation Agency Vinnova, 2019-02261, and the EU (FP7) framework program, for the financial support of DOPPLER-CIP project (grant number 223615).

## Author contributions

**Tino Ebbers:** Writing – original draft, Conceptualization, Writing – review and editing. **Jan Engvall:** Writing – review and editing, Investigation. **Ann Bolger:** Writing – review and editing. **Mariana Bustamante:** Methodology, Writing – review and editing. **Federica Viola:** Visualization, Software, Methodology, Conceptualization, Writing – original draft, Writing – review and editing.

## Ethics approval and consent

The research was performed in line with the Declaration of Helsinki and was approved by the regional ethics board. The exams were performed specifically for research purposes between 2011 and 2015 and included retrospectively in this study.

## Consent for publication

All subjects gave written informed consent to participate in the study.

## Declaration of competing interests

The authors declare that they have no known competing financial interests or personal relationships that could have appeared to influence the work reported in this paper.
